# Associations of Eating Habits with Self-Rated Health and Life Satisfaction in Adolescents: A 42-Country Cross-Sectional Study

**DOI:** 10.3390/ejihpe14060099

**Published:** 2024-05-23

**Authors:** Sitong Chen, José Francisco López-Gil, Aamir Raoof Memon, Ran Bao, Xingyi Yang

**Affiliations:** 1 Institute for Health and Sport, Victoria University, Melbourne, VIC 3011, Australia; aamir.raoof@live.vu.edu.au; 2 One Health Research Group, Universidad de Las Américas, Quito 170124, Ecuador; 3 Centre for Active Living and Learning, University of Newcastle, Callaghan, NSW 2308, Australia; ran.bao@uon.edu.au; 4 School of Education, College of Human and Social Futures, University of Newcastle, Callaghan, NSW 2308, Australia; 5 Active Living Research Program, Hunter Medical Research Institute, New Lambton Heights, NSW 2305, Australia; 6 Centre for Mental Health, Shenzhen University, Shenzhen 518060, China; yangxingyi1208@gmail.com

**Keywords:** self-rated health, life satisfaction, breakfast, fruit, vegetables, sweets, soft drinks

## Abstract

Objective: The aim of this study was to explore the associations of eating habits with self-rated health and life satisfaction in adolescents using a multiple-country sample. Methods: Cross-sectional data from the Health Behavior in School-Aged Children (HBSC) 2013/2014 wave was used in this study. A self-reported questionnaire was used to collect data on independent variables including breakfast on weekdays, breakfast at weekends, and consumption of fruits, vegetables, sweets, and soft dirks. Outcomes included self-rated health and life satisfaction. Regression models were used to assess the associations between the independent variables and the two outcomes, separately, after controlling for covariates. Results were presented using odds ratios (ORs) and 95% confidence intervals (CIs). Results: Of all the study participants (aged 11–15 years), 50.8% were girls. Compared with no consumption of breakfast on weekdays, eating breakfast for five days had 1.22 times greater likelihood for improved self-rated health (OR = 1.22, 95% CI: 1.19–1.25, *p* < 0.001). Participants who ate breakfast for both days (OR = 1.41, 95% CI: 1.36–1.46, *p* < 0.001) and one day (OR = 1.12, 95% CI = 1.08–1.17, *p* < 0.001) were more likely to experience improved self-rated health compared to never eating breakfast at weekends. Five or more days for fruit and vegetable consumption resulted in better self-rated health (all *p* < 0.001). Similar results were found in terms of the associations of breakfast, fruit, and vegetable consumption with life satisfaction. For example, a higher frequency of fruit intake was associated with enhanced self-rated health (e.g., OR for more than once daily = 1.42, 95% CI: 1.34–1.51, *p* < 0.001) compared to no fruit consumption. Similarly, a higher-frequency vegetable intake, such as more than once daily (OR = 1.33, 95% CI: 1.26–1.39, *p* < 0.001), was associated with improved self-rated health. Conclusions: Healthy eating habits, especially regular breakfast and a higher consumption of vegetables and fruit, are associated with better self-rated health and life satisfaction in school-aged children. Of note, the consumption of fruit would have the greatest impact on health and wellbeing outcomes. This study offers evidence that healthy eating habits can play a vital role in school-aged children’s health and wellbeing, highlighting the practical significance of educating adolescents to develop healthy eating habits.

## 1. Introduction

Global trends have shown a substantial decrease in health outcomes (e.g., physical, emotional, and mental) among children in recent decades [1,2]. For example, a national survey found that US children’s mental challenges have increased by over 20% from 2016 to 2019 [3]. In addition, the prevalence of poor self-rated health has grown over the past 14 years among adolescents [4]. Given that health problems during childhood may carry forward into adulthood and have a long-lasting effect on an individual’s achievement and life outcomes [5], several studies have thus attempted to identify the range of factors contributing to this emerging public health issue [6,7,8,9].

In recent years, the burgeoning field of nutritional psychiatry has brought to our attention the effects of eating habits (as a modifiable factor) on an individual’s health and wellbeing [10]. For example, two meta-analyses showed that an increased intake of a healthy diet (e.g., a high intake of fruits and vegetables) was associated with lower risks for mental health issues [11,12]. Given that eating habits and nutritional needs vary across a person’s lifespan, these patterns may not be similar among children. For instance, children show a higher preference for sweet foods than adults [13], which may place them at greater risk of poor growth and development outcomes [14]. However, studies examining the association between eating habits and children’s self-rated health are lacking [15,16]. From a global perspective, little is known about the effect of multiple eating habits on children’s different dimensions of health, which leads to a barrier for future prevention and intervention programs.

Self-rated health, as an important dimension of children’s health and wellbeing, may be related to eating habits, including healthy food consumption (e.g., fruits and vegetables), healthy dietary behaviours (e.g., regular breakfast consumption), and unhealthy food consumption (e.g., sugar-sweetened beverages, and soft drinks). For example, previous studies have indicated that fruits and vegetables, which contain rich sources of vitamins, dietary fibre, and minerals [17], are strongly linked to a lower risk of many diseases, such as cardiovascular disease [18,19], cancer [18,19] and type 2 diabetes [20]. Therefore, most nutritional guidelines recommend consuming at least 1 to 1.5 cups of fruits and 1 to 1.5 cups of vegetable intake per day [21]. Moreover, the importance of regular breakfast on children’s health outcomes has also been highlighted in several studies [12,22,23,24]. On the other hand, a high consumption of sweets and soft drinks is associated with adverse health outcomes, including cardiovascular disease, overweight [25], type 2 diabetes [26], and metabolic syndrome [27]. There is also emerging evidence of an association between junk food consumption and cancer [28]. However, it is necessary to note that many of those studies have focused on physical diseases. As such, the effect of multiple eating habits on children’s self-rated health—a comprehensive measure of health status—has not yet been extensively explored.

Health and wellbeing also include domains related to children’s positive psychological functioning. There is a general assumption that having healthy eating habits and maintaining life satisfaction is paramount for children; however, the role of eating habits on positive psychological outcomes among children is currently underexplored [29,30]. Certain studies have found that children who consumed five or more portions of fruit and vegetables had 3.73 times higher scores in well-being than those not eating any fruit and vegetables [31]. This is consistent with a study on breakfast consumption, which found that a higher frequency of breakfast eating was associated with higher happiness scores among 140 high school students [32]. Interestingly, another study including 527 Spanish adolescents provides contradictory findings, indicating that those who skipped breakfast regularly showed better psychological well-being than breakfast-eaters [33]. These findings are preliminary, given the limitations in measurement of breakfast frequency (e.g., the difference between weekday and weekend was neglected) in these studies. Thus, a large-scale study for a comprehensive exploration of eating habits is needed.

The findings between the consumption of unhealthy food and an individual’s psychological outcomes are also mixed. People generally assume that “unhealthy food brings a good mood” [34]. Limited evidence supports this and suggests that soft drinks may decrease children’s feelings of unhappiness [35]. However, information on the unhappiness level of children was obtained from their mothers using a single item with three response options. The likelihood of bias in case of single-item measures as well as responses obtained from others, rather than the participants, has been widely recognized [36,37]. Conversely, there are studies showing that junk food (e.g., sugar products) may negatively impact children’s mental health [38,39]. The theory of positive psychology points out that positive psychosocial outcomes implying the absence of mental problems are not simple [40]. Another research gap is that the associations between multiple eating habits and different dimensions of health and wellbeing indicators remain understudied. This suggests that the association between eating habits and children’s positive psychological outcomes still needs to be elucidated. Therefore, the aim of this study was to explore the association between eating habits and different dimensions of health and wellbeing (i.e., self-rated health and life satisfaction) using a representative sample of adolescents from different countries. This study uses a multi-country sample to examine the association between eating habits and life satisfaction, which can enhance the generalization of study findings and provide stronger evidence base to support improving eating habits. Also, this study will consider a series of eating habits, and the findings can provide a more comprehensive understanding of eating habits and their role in health and wellbeing.

## 2. Method

### 2.1. Study Design

Cross-sectional data from the 2013/2014 Health Behaviour in School-Aged Children (HBSC) survey (started in September 2013 and ended in June 2014) was used in this study. The HBSC research network is an international alliance of researchers that collaborate on the cross-national surveying of school students. The survey aims to assess health behaviours (e.g., physical activity, eating habits), risk behaviours (e.g., smoking, alcohol use), weight control and body image (e.g., body mass), violence and injuries (e.g., severity), family culture (e.g., family support), peer relationship (e.g., peer support), health and wellbeing (e.g., self-rated health), school setting (e.g., school engagement), social inequality (e.g., family socioeconomic level), and puberty. The HBSC collected data on adolescents’ health and well-being, their social environments, and health behaviours. The specific population selected for sampling is young people attending school aged 11, 13, and 15, with each country selecting a nationally randomized representative sample of school-aged students. Participants are recruited by randomly selecting classes within targeted school years/grades. In most cases, only one class was selected for each grade/year, but there was more than one on some occasions (e.g., if class sizes were small). The inclusion criterion for participants covered all students in the relevant classes in the participating schools; the exclusion criteria covered students absent from school on the day of data collection, students who did not want to participate, and students whose parents rejected their children’s participation. In total, 214,080 participants from 42 countries were included in this study. More information about the HBSC can be found at www.hbsc.org. For the 2013/2014 survey, the study protocol can be accessed at https://hbsc.org/publications/survey-protocols/ (accessed on 30 November 2023). Informed consent was obtained from the children involved in research projects, as well as their parents or guardians.

### 2.2. Measures

(1) Exposure: eating habits

The following eating habits were assessed: breakfast on weekdays and weekends, fruits, vegetables, sweets, and soft drinks. Breakfast on weekdays and weekends were investigated by asking the question “How often do you usually have breakfast (more than a glass of milk or fruit juice)?” Options for weekdays ranged from never to 5 days, and those for weekends ranged from never to both days. The frequencies of fruit, vegetables, sweets, and soft drink intake were established by the question “How many times a week do you usually eat or drink fruits/vegetables/sweets (candy or chocolate)/coke or other soft drinks that contain sugar?” Response options ranged from never to more than once daily. The above measures were based on the Food Frequency Questionnaire, which has shown good psychometric properties, with reliability ranging from 0.43 to 0.70 and validity ranging from 0.10 to 0.65 [41].

(2) Outcomes: self-rated health and life satisfaction

The options for self-rated health were as follows; poor, fair, good, and excellent, with the reliability of Cronbach’s alpha being 0.80 in boys and 0.84 in girls [42]. Life satisfaction was measured by giving participants a picture of a ladder. Participants indicated their current life satisfaction by choosing one position on the ladder from 0 (worst possible life) to 10 (best possible life). This measure has been validated in previous studies, with reliability ranging from 0.66 and 0.70 and validity ranging from 0.42 to 0.56 [43].

(3) Covariates

Based on the previous studies considering the following factors as covariates [44,45,46,47,48], the present study also included sex (girls/boys), age (11/13/15 years), if the family was well-off (very/quite/average/not very/not at all), body mass index (BMI), physical activity, TV watching, playing computer games, computer usage, smoking, and alcohol consumption. These were assessed and treated as covariates in the subsequent statistical analyses. Participants’ BMI was calculated based on self-reported height and weight, using the formular kg (weight)/m^2^ (height). Physical activity was assessed by inquiring about the frequency of being physically active for at least 60 min per day in the past week (responses: 0–7 days). Daily hours of TV watching, playing computer games, and computer usage on weekdays and weekends were assessed, ranging from “none at all” to “7 h or more a day”. The frequency of current smoking ranged from “every day” to “don’t”. The frequency of alcohol use in the last 30 days ranged from “never” to “30 days or more”. Headache, stomach ache, backache, feeling low, irritability or bad temper, feeling nervous, difficulties in sleeping, and feeling dizzy were also considered as covariates. The frequency of each above variable ranged from “about every day” (coded as 1) to “rarely or never” (coded as 5).

### 2.3. Statistical Analysis

All the statistical analyses were performed using STATA BE 18.0 (College Station, TX, USA). Multiple imputation was used to cope with the missing cases of variables of interest. Descriptive statistics were used to report sample characteristics. Generalized linear models were used to assess the associations of eating habits with self-rated health and life satisfaction, while controlling for all the covariates. Two models were ultimately developed: (1) model 1 was used to assess the association between eating habits (the highest level of the indicators as reference group) and self-rated health, using ordinal logistic regression; (2) model 2 was used to assess the association between eating habits (the highest level of the indicators as reference group) and life satisfaction, using ordinal logistic regression. Results from model 1 were presented as an odds ratio (OR) with 95% confidence interval (CI), and results from model 2 were presented as beta with 95% CI. Sex- and age-stratified analyses were repeatedly conducted as well. A *p*-value below 0.05 was used to ascertain statistical significance.

## 3. Results

### 3.1. Sample Characteristics

The sample characteristics are shown in Table 1. Of the 214,080 participants aged 11–15 years, around half were girls. Of the total sample, more than 63% reported eating breakfast for five days. At weekends, the percentage who ate breakfast for two days was approximately 80%. In addition, one in five adolescents ate fruits more than once daily; 16.6% and 19.0% of adolescents reported “more than once daily” and “once daily”, respectively. The percentage of adolescents who reported eating sweets more than once daily was around 12%. One in ten adolescents had soft drinks more than once daily. In addition, extremely few adolescents rated their health as excellent; around 12% of them reported good self-rated health. The mean of raw scores of life satisfaction was 7.6. More details on sample characteristics can be found in Table 1.

### 3.2. Associations between Eating Habits and Self-Rated Health

Table 2 presents the results from logistic regression analyses on the association between eating habits and self-rated health. Results indicated that a higher frequency of breakfast consumption on weekdays and weekend days was associated with greater self-rated health. For example, compared with no consumption of breakfast on weekdays, eating breakfast for five days had a 1.22 times greater likelihood of improved self-rated health. Participants who ate breakfast for both days and one day were more likely to experience improved self-rated health compared to never eating breakfast at weekends. In addition, a higher frequency of fruit intake was associated with enhanced self-rated health compared to no fruit consumption. Similarly, a higher frequency of vegetable intake, such as more than once daily, was associated with improved self-rated health. Interestingly, never eating sweets or eating them less than once a week or once a week was more likely to result in enhanced self-rated health. Surprisingly, a lower frequency of consuming soft drinks was less likely to be associated with improved self-rated health. Results on the subgroup analysis can be found in Tables S1–S5 (Supplementary Materials).

### 3.3. Associations between Eating Habits and Life Satisfaction

The results regarding the association between eating habits and life satisfaction are displayed in Table 3. A higher frequency of breakfast on weekdays, breakfast at weekends, and intake of fruits and vegetables were all associated with higher life satisfaction scores. Specifically, eating breakfast for five days on weekdays and/or both days at weekends was positively associated with life satisfaction. A higher frequency of fruit consumption, such as more than once daily, was related to greater life satisfaction. Similarly, participants who ate vegetables more than once daily were more likely to report improved life satisfaction. Additionally, a lower consumption of sweets and soft drinks was negatively associated with life satisfaction. Results on the subgroup analysis can be found in Tables S6–S10 (Supplementary Materials).

## 4. Discussion

This study broadens research insights into factors of health and wellbeing in school-aged children, using a large sample based on the HBSC 2013/2014 survey round. In summary, our results mainly indicated that a lower frequency of consumption of breakfast, fruits, and vegetables was associated with poorer self-rated health. Conversely, a higher frequency of breakfast consumption and fruit and vegetable intake was positively associated with better life satisfaction. Such associations were consistent across sex and age groups. Given that our sample was multiple-country and representative to each single included country, it is credible that the findings of this study are highly generalized and could be used in a wider range of settings.

To assess health and wellbeing comprehensively, two outcomes (i.e., self-rated health and life satisfaction) were assessed in our study. Self-rated health is an important indicator of health research, which refers to a subjective and general perception of individuals’ health [49,50]. Convincing evidence has shown the significance of self-rated health in predicting objectively assessed health outcomes [49,50], and there is reliable evidence supporting breakfast [23,51], fruit, and vegetable consumption as being positively associated with better self-rated health in adolescents [52,53,54], which is consistent with our research findings. This consistency highlights the importance of having regular breakfast-eating and higher fruit and vegetable consumption habits to promote self-rated health, thereby potentially enhancing the health and wellbeing of school-aged children. However, frequent skipping of breakfast and a tendency to decline fruit and vegetables have been observed in school-aged children [24,55,56,57]. These health concerns may be barriers to promote self-rated health, which should be addressed by effective nutritional education and dietary intervention plans.

Our results indicate that regular breakfast and more fruit and vegetable consumption could increase life satisfaction in school-aged children. To our knowledge, little is known about the association between breakfast consumption and life satisfaction in school-aged children. In this regard, the current study advances the existing knowledge. We could use these results to explain this association, as breakfast was associated with higher self-rated health in adolescents, thereby probably promoting life satisfaction [58,59]. Such a reasonable interpretation is merely based on research results, but more evidence should be accumulated for better clarification. This implies that more relevant research is needed in the future. Moreover, sufficient evidence based on correlation, longitudinal, and experimental studies has supported the positive role of fruits and vegetables on enhanced life satisfaction [60,61]. These previous studies can corroborate the present study, further emphasising the significant health impact of eating more fruits and vegetables for school-aged children. As we discussed before, owing to declining trends in fruit and vegetable consumptions, it is necessary to reverse this change to improve school-aged children’s health and wellbeing.

In the examination of the associations between eating habits and health outcomes, our study revealed small effect sizes. The small effect size observed in our study is reflective of the complex nature of eating habits’ influence on health and wellbeing. Furthermore, the utility of recognizing small effect sizes lies in highlighting the importance of even modest improvements in eating habits, which can lead to significant public health benefits at the population level. Thus, despite the small effect size found in our study, the implications for public health policy and individual dietary recommendations remain meaningful.

According to current knowledge, the underlying mechanisms linking food intake (e.g., fruits, vegetables, sweets) and health and wellbeing are not yet clear [29]. Despite this, some plausible explanations can be used to better understand the research findings in the current study. Specifically, the rich array of vitamins, minerals, and antioxidants found in fruits and vegetables plays a crucial role in mitigating oxidative stress and inflammation, processes known to affect mood and overall well-being negatively. For instance, antioxidants like vitamin C and flavonoids can cross the blood–brain barrier, offering neuroprotective effects that improve mood and cognitive function [62,63]. Secondly, the dietary fibres present in these foods regulate gut health, which is closely linked to mental health through the gut–brain axis. A healthy gut microbiome is associated with lower levels of stress, anxiety, and depression, thereby contributing to higher life satisfaction [64]. Moreover, starting the day with a balanced breakfast has been shown to stabilize blood sugar levels throughout the day, preventing the energy dips and mood fluctuations that can detract from life satisfaction [65]. This meal sets the metabolic tone for the day, impacting concentration, energy levels, and the ability to engage in daily activities with a positive mindset [66]. Collectively, these dietary practices not only support physical health but also foster a sense of well-being and satisfaction with life by influencing biochemical pathways, psychological states, and daily energy and mood patterns. Several factors can influence the efficacy of these mechanisms, such as environmental factors (e.g., access to fresh produce) and socioeconomic status, and stress management techniques can modulate these effects [33]. The mechanism should be elucidated in the future, but this uncertainty does not call into doubt the trustworthiness of evidence on eating habits and wellbeing in adolescents. Despite the uncertainty regarding the mechanisms on how better eating habits enhance wellbeing, the current study can provide some practical implications for wellbeing promotion. For example, not skipping any breakfast would be a “morning investment” and higher fruit and vegetables consumption is recommended for better health and wellbeing in school-aged children.

### 4.1. Study Strengths and Limitations

This study has some strengths, including the large sample size and the multiple eating behaviours examined. These two strengths can help increase the research generalizability and specify the associations between different eating behaviours and health-related outcomes, respectively. However, some study limitations should be mentioned. First, the cross-sectional study design that this study used cannot infer causal association between the exposure and outcomes. The true impacts of eating habits on health and wellbeing indicators should be further explored by studies at the micro-level. Second, all the measurements in this study were self-reported, which may negatively influence the measurement accuracy and lead to measurement errors. Third, the measures of health and wellbeing were based on study participants’ perceptions toward their overall health. Self-reported measures might be a barrier to accurately understand the association between eating habits and health and wellbeing indicators. Fourth, the current study cannot provide more information of confounders on health and wellbeing, such as medical conditions, which could be a factor influencing our research findings. The current study can only analyse data on eating habits in terms of quantity instead of quality. As is known, the quality of food intake also plays a role in promoting health and wellbeing [29], but it was beyond the scope of this study. As the data this study used were collected between 2013 and 2014, the findings of this study may not totally reflect the current situation, given lifestyle changes in children and adolescents over the past decade. Care must be taken if the findings are used to guide current practice. In sum, future studies should address the limitations for a robust evidence base on eating habits and health and wellbeing in school-aged children.

### 4.2. Implications for Practice and Research

Based on the findings observed in this study, some implications for practice and research can be discussed. In order to improve self-rated health and life satisfaction, ensuring daily regular breakfast and increasing the consumption of vegetables and fruit are essential approaches; in the meantime, limiting the intake of sweets as much as possible is also necessary. These joint practices in eating habits may play important roles in enhancing health and wellbeing in adolescents. In addition to practice, owing to the unclear mechanism linking eating habits and health and wellbeing indicators, future research is supported to explore how eating habits truly impact self-rated health and life satisfaction. in children and adolescents caused by motorization, industrialization, and the COVID-19 pandemic over the past decade. However, studies conducted during the COVID-19 pandemic found that healthy eating habits, such as a higher intake of vegetables and fruit, were positively related to health and wellbeing indicators in adolescents [67,68]. These findings observed from the COVID-19 studies can support our study, which further corroborates that the associations discovered in this study may not be significantly affected. Despite this, caution must be taken if the findings are used to guide current practice and policy.

## 5. Conclusions

Findings of this study suggest that healthy eating habits (e.g., regular breakfast, higher consumption of vegetables and fruit) are favourably associated with higher self-rated health and life satisfaction. This study also offers evidence that optimal eating habits can play a crucial role in promoting health and wellbeing in adolescents. Based on the findings, it is recommended that to promote better wellbeing for adolescents’ development and growth, improving daily breakfast and fruit and vegetable consumption would bring certain positive effects.

## Figures and Tables

**Table 1:** Sample characteristic of this study (*n* = 214,080).

Categorical Variables		Proportion (%)	
Sex	Boys	49.2	
	Girls	50.8	
Age (years)	11	32.2	
	13	34.6	
	15	33.2	
Family well-off	Very	19.5	
	Quite	34.4	
	Average	39.0	
	Not very	5.7	
	Not at all	1.4	
Breakfast on weekdays	Never	15.7	
	One day	4.0	
	Two days	4.9	
	Three days	6.6	
	Four days	5.5	
	Five days	63.3	
Breakfast at weekends	Never	7.1	
	One day	13.3	
	Both days	79.5	
Fruit consumption	Never	3.0	
	Less than once a week	6.0	
	Once a week	9.5	
	2–4 days a week	28.2	
	5–6 days a week	15.4	
	Once daily	17.7	
	More than once daily	20.2	
Vegetable consumption	Never	4.5	
	Less than once a week	5.8	
	Once a week	9.8	
	2–4 days a week	25.3	
	5–6 days a week	19.1	
	Once daily	19.0	
	More than once daily	16.6	
Sweet consumption	Never	4.1	
	Less than once a week	12.1	
	Once a week	19.2	
	2–4 days a week	28.2	
	5–6 days a week	12.8	
	Once daily	12.1	
	More than once daily	11.6	
Soft drink consumption	Never	11.1	
	Less than once a week	22.0	
	Once a week	18.8	
	2–4 days a week	21.7	
	5–6 days a week	8.7	
	Once daily	7.4	
	More than once daily	10.4	
Self-rated health	Excellent	1.6	
	Good	11.8	
	Fair	50.2	
	Poor	36.4	
Continuous variables	Mean	95% CI	
Body mass index	19.53	19.51	19.54
Life satisfaction	7.63	7.62	7.64

**Table 2:** Results of the ordinal logistic model using self-rated health as the outcome.

	OR	95% CI		*p* Value
Breakfast on weekdays				
Never	Reference			
One day	1.04	1.00	1.10	0.078
Two days	0.98	0.94	1.03	0.504
Three days	0.96	0.92	1.00	0.039
Four days	1.00	0.95	1.04	0.856
Five days	1.22	1.19	1.25	<0.001
Breakfast at weekends				
Never	Reference			
One day	1.12	1.08	1.17	<0.001
Both days	1.41	1.36	1.46	<0.001
Fruit consumption				
Never	Reference			
Less than once a week	0.88	0.83	0.94	<0.001
Once a week	0.98	0.92	1.04	0.430
2–4 days a week	1.06	1.00	1.12	0.056
5–6 days a week	1.17	1.11	1.24	<0.001
Once daily	1.29	1.22	1.37	<0.001
More than once daily	1.42	1.34	1.51	<0.001
Vegetable consumption				
Never	Reference			
Less than once a week	0.97	0.91	1.02	0.223
Once a week	1.05	0.99	1.10	0.089
2–4 days a week	1.10	1.05	1.15	<0.001
5–6 days a week	1.16	1.10	1.21	<0.001
Once daily	1.21	1.15	1.27	<0.001
More than once daily	1.33	1.26	1.39	<0.001
Sweet consumption				
Never	1.13	1.07	1.19	<0.001
Less than once a week	1.11	1.07	1.16	<0.001
Once a week	1.07	1.04	1.11	<0.001
2–4 days a week	1.00	0.96	1.03	0.793
5–6 days a week	0.98	0.95	1.02	0.393
Once daily	1.03	0.99	1.06	0.157
More than once daily	Reference			
Soft drink consumption				
Never	0.82	0.79	0.86	<0.001
Less than once a week	0.83	0.80	0.86	<0.001
Once a week	0.85	0.82	0.88	<0.001
2–4 days a week	0.85	0.82	0.88	<0.001
5–6 days a week	0.88	0.84	0.92	<0.001
Once daily	0.95	0.91	0.99	0.011
More than once daily	Reference			

Note: Models were adjusted for sex, age, if the family was well-off, body mass index, physical activity, TV watching, playing computer games, computer use, smoking, and alcohol intake. OR: odds ratio; CI: confidence interval; Reference: reference group.

**Table 3:** Results of linear regression analyses using life satisfaction as outcome (higher scores indicate higher life satisfaction).

	Coefficient	95% CI		*p* Value
Breakfast on weekdays				
Never	Reference			
One day	0.03	−0.01	0.08	0.151
Two days	0.02	−0.02	0.07	0.249
Three days	0.04	0.00	0.07	0.049
Four days	0.04	0.00	0.08	0.061
Five days	0.25	0.22	0.27	<0.001
Breakfast at weekends				
Never	Reference			
One day	0.16	0.12	0.19	<0.001
Both days	0.36	0.32	0.39	<0.001
Fruit consumption				
Never	Reference			
Less than once a week	−0.11	−0.16	−0.05	<0.001
Once a week	0.01	−0.04	0.07	0.642
2–4 days a week	0.07	0.02	0.12	0.011
5–6 days a week	0.15	0.10	0.20	<0.001
Once daily	0.19	0.14	0.24	<0.001
More than once daily	0.27	0.21	0.32	<0.001
Vegetable consumption				
Never	Reference			
Less than once a week	0.06	0.01	0.11	0.027
Once a week	0.08	0.04	0.13	<0.001
2–4 days a week	0.12	0.07	0.16	<0.001
5–6 days a week	0.14	0.10	0.18	<0.001
Once daily	0.13	0.09	0.18	<0.001
More than once daily	0.15	0.11	0.20	<0.001
Sweet consumption				
Never	−0.07	−0.12	−0.03	0.003
Less than once a week	−0.05	−0.09	−0.02	0.002
Once a week	−0.03	−0.07	0.00	0.036
2–4 days a week	−0.06	−0.09	−0.03	<0.001
5–6 days a week	−0.05	−0.08	−0.01	0.005
Once daily	−0.01	−0.04	0.02	0.594
More than once daily	Reference			
Soft drink consumption				
Never	−0.07	−0.11	−0.03	<0.001
Less than once a week	−0.09	−0.12	−0.06	<0.001
Once a week	−0.07	−0.10	−0.03	<0.001
2–4 days a week	−0.09	−0.12	−0.06	<0.001
5–6 days a week	−0.06	−0.10	−0.03	0.001
Once daily	−0.02	−0.06	0.02	0.395
More than once daily	Reference			

Note: Models were adjusted for sex, age, if the family was well-off, body mass index, physical activity, TV watching, playing computer games, computer use, smoking, and alcohol intake. CI: confidence interval. Reference: reference group.

## Data Availability

Data is contained within the article.

## Supplementary Materials

The following supporting information can be downloaded at: https://www.mdpi.com/article/10.3390/ejihpe14060099/s1.
