# Supplementary material for: Associations of Eating Habits with Self-Rated Health and Life Satisfaction in Adolescents: A 42-Country Cross-Sectional Study
Source: Eur J Investig Health Psychol Educ. 2024 May 23;14(6):1501–13. doi: 10.3390/ejihpe14060099 (PMC11202602; doi:10.3390/ejihpe14060099)
Supplement: Supplementary file 1 [file ejihpe-14-00099-s001.zip › ejihpe-2864631-supplementary.pdf]

Table S1. Results of the ordinal logistic model using self-rated health as the outcome in boys.

	OR	95% CI		*P value*
**Breakfast on weekdays**				
Never	Reference			
One day	1.03	1.02	1.04	<0.001
Two days	0.97	0.96	0.98	<0.001
Three days	0.92	0.91	0.92	<0.001
Four days	0.99	0.98	1.00	0.004
Five days	1.17	1.17	1.18	<0.001
**Breakfast at weekends**				
Never	Reference			
One day	1.08	1.07	1.08	<0.001
Both days	1.32	1.31	1.33	<0.001
**Fruit consumption**				
Never	Reference			
Less than once a week	0.88	0.87	0.89	<0.001
Once a week	1.03	1.02	1.04	<0.001
2-4 days a week	1.08	1.07	1.09	<0.001
5-6 days a week	1.21	1.20	1.23	<0.001
Once daily	1.30	1.29	1.31	<0.001
More than once daily	1.51	1.50	1.53	<0.001
**Vegetable consumption**				
Never	Reference			
Less than once a week	0.91	0.90	0.92	<0.001
Once a week	0.99	0.98	1.00	0.083
2-4 days a week	1.03	1.02	1.04	<0.001
5-6 days a week	1.10	1.09	1.11	<0.001
Once daily	1.13	1.12	1.14	<0.001
More than once daily	1.22	1.21	1.23	<0.001
**Sweet consumption**				
Never	1.17	1.15	1.18	<0.001
Less than once a week	1.06	1.05	1.06	<0.001
Once a week	1.02	1.02	1.03	<0.001
2-4 days a week	0.94	0.94	0.95	<0.001
5-6 days a week	0.92	0.92	0.93	<0.001
Once daily	0.98	0.97	0.99	<0.001
More than once daily	Reference			
**Soft drink consumption**				
Never	0.84	0.83	0.84	<0.001
Less than once a week	0.83	0.83	0.84	<0.001
Once a week	0.86	0.86	0.87	<0.001
2-4 days a week	0.85	0.84	0.85	<0.001
5-6 days a week	0.88	0.87	0.88	<0.001
Once daily	0.94	0.94	0.95	<0.001
More than once daily	Reference			

Note: Models were adjusted for sex, age, if the family was well-off, body mass index, physical activity, TV watching, playing computer games, computer use, smoking, and alcohol intake. OR: odds ratio; CI: confidence interval; Reference: reference group.

Table S2. Results of the ordinal logistic model using self-rated health as the outcome in girls.

	OR	95% CI		*P value*
**Breakfast on weekdays**				
Never	Reference			
One day	1.05	1.04	1.05	<0.001
Two days	0.99	0.98	1.00	0.002
Three days	0.98	0.98	0.99	<0.001
Four days	0.99	0.98	1.00	0.008
Five days	1.24	1.23	1.25	<0.001
**Breakfast at weekends**				
Never	Reference			
One day	1.18	1.17	1.19	<0.001
Both days	1.51	1.50	1.52	<0.001
**Fruit consumption**				
Never	Reference			
Less than once a week	0.89	0.88	0.90	<0.001
Once a week	0.93	0.91	0.94	<0.001
2-4 days a week	1.04	1.03	1.05	<0.001
5-6 days a week	1.14	1.12	1.15	<0.001
Once daily	1.27	1.25	1.29	<0.001
More than once daily	1.35	1.34	1.37	<0.001
**Vegetable consumption**				
Never	Reference			
Less than once a week	1.06	1.04	1.07	<0.001
Once a week	1.12	1.11	1.13	<0.001
2-4 days a week	1.19	1.18	1.20	<0.001
5-6 days a week	1.25	1.24	1.26	<0.001
Once daily	1.34	1.32	1.35	<0.001
More than once daily	1.47	1.46	1.49	<0.001
**Sweet consumption**				
Never	1.06	1.05	1.07	<0.001
Less than once a week	1.16	1.15	1.17	<0.001
Once a week	1.12	1.11	1.13	<0.001
2-4 days a week	1.05	1.04	1.05	<0.001
5-6 days a week	1.04	1.04	1.05	<0.001
Once daily	1.07	1.06	1.08	<0.001
More than once daily	Reference			
**Soft drink consumption**				
Never	0.84	0.84	0.85	<0.001
Less than once a week	0.85	0.85	0.86	<0.001
Once a week	0.87	0.86	0.88	<0.001
2-4 days a week	0.88	0.88	0.89	<0.001
5-6 days a week	0.90	0.89	0.91	<0.001
Once daily	0.96	0.95	0.97	<0.001
More than once daily	Reference			

Note: Models were adjusted for sex, age, if the family was well-off, body mass index, physical activity, TV watching, playing computer games, computer use, smoking, and alcohol intake. OR: odds ratio; CI: confidence interval; Reference: reference group.

Table S3. Results of the ordinal logistic model using self-rated health as the outcome in 11-year-olds.

	OR	95% CI		*P value*
**Breakfast on weekdays**				
Never	Reference			
One day	1.10	1.08	1.11	<0.001
Two days	0.95	0.93	0.96	<0.001
Three days	0.93	0.92	0.94	<0.001
Four days	0.97	0.96	0.98	<0.001
Five days	1.18	1.18	1.19	<0.001
**Breakfast at weekends**				
Never	Reference			
One day	1.01	1.00	1.02	0.037
Both days	1.25	1.24	1.27	<0.001
**Fruit consumption**				
Never	Reference			
Less than once a week	0.99	0.97	1.00	0.071
Once a week	1.06	1.04	1.07	<0.001
2-4 days a week	1.15	1.14	1.17	<0.001
5-6 days a week	1.23	1.21	1.25	<0.001
Once daily	1.44	1.42	1.46	<0.001
More than once daily	1.59	1.57	1.61	<0.001
**Vegetable consumption**				
Never	Reference			
Less than once a week	0.94	0.93	0.95	<0.001
Once a week	1.01	1.00	1.02	0.039
2-4 days a week	1.08	1.07	1.09	<0.001
5-6 days a week	1.17	1.16	1.18	<0.001
Once daily	1.26	1.25	1.28	<0.001
More than once daily	1.37	1.36	1.39	<0.001
**Sweet consumption**				
Never	1.19	1.18	1.21	<0.001
Less than once a week	1.19	1.18	1.20	<0.001
Once a week	1.12	1.11	1.13	<0.001
2-4 days a week	1.05	1.04	1.05	<0.001
5-6 days a week	1.02	1.01	1.03	<0.001
Once daily	1.01	1.00	1.02	0.025
More than once daily	Reference			
**Soft drink consumption**				
Never	0.89	0.88	0.90	<0.001
Less than once a week	0.85	0.85	0.86	<0.001
Once a week	0.88	0.87	0.89	<0.001
2-4 days a week	0.85	0.84	0.86	<0.001
5-6 days a week	0.92	0.91	0.93	<0.001
Once daily	0.93	0.92	0.94	<0.001
More than once daily	Reference			

Note: Models were adjusted for sex, age, if the family was well-off, body mass index, physical activity, TV watching, playing computer games, computer use, smoking, and alcohol intake. OR: odds ratio; CI: confidence interval; Reference: reference group.

Table S4. Results of the ordinal logistic model using self-rated health as the outcome in 13-year-olds.

	**OR**	**95% CI**		***P value***
**Breakfast on weekdays**				
Never	Reference			
One day	1.04	1.03	1.05	<0.001
Two days	1.00	0.99	1.01	0.749
Three days	0.94	0.93	0.95	<0.001
Four days	0.96	0.95	0.97	<0.001
Five days	1.20	1.20	1.21	<0.001
**Breakfast at weekends**				
Never	Reference			
One day	1.19	1.17	1.20	<0.001
Both days	1.50	1.49	1.51	<0.001
**Fruit consumption**				
Never	Reference			
Less than once a week	0.77	0.76	0.78	<0.001
Once a week	0.89	0.88	0.91	<0.001
2-4 days a week	0.94	0.93	0.95	<0.001
5-6 days a week	1.06	1.04	1.07	<0.001
Once daily	1.13	1.12	1.15	<0.001
More than once daily	1.27	1.26	1.29	<0.001
**Vegetable consumption**				
Never	Reference			
Less than once a week	1.03	1.02	1.04	<0.001
Once a week	1.13	1.11	1.14	<0.001
2-4 days a week	1.17	1.16	1.18	<0.001
5-6 days a week	1.22	1.21	1.23	<0.001
Once daily	1.29	1.27	1.30	<0.001
More than once daily	1.40	1.38	1.41	<0.001
**Sweet consumption**				
Never	1.09	1.08	1.11	<0.001
Less than once a week	1.05	1.04	1.05	<0.001
Once a week	1.03	1.02	1.03	<0.001
2-4 days a week	0.95	0.95	0.96	<0.001
5-6 days a week	0.96	0.95	0.97	<0.001
Once daily	1.02	1.01	1.03	<0.001
More than once daily	Reference			
**Soft drink consumption**				
Never	0.79	0.78	0.80	<0.001
Less than once a week	0.80	0.79	0.81	<0.001
Once a week	0.82	0.82	0.83	<0.001
2-4 days a week	0.82	0.81	0.82	<0.001
5-6 days a week	0.84	0.83	0.85	<0.001
Once daily	0.93	0.92	0.94	<0.001
More than once daily	Reference			

Note: Models were adjusted for sex, age, if the family was well-off, body mass index, physical activity, TV watching, playing computer games, computer use, smoking, and alcohol intake. OR: odds ratio; CI: confidence interval; Reference: reference group.

Table S5. Results of the ordinal logistic model using self-rated health as the outcome in 15-year-olds.

	OR	95% CI		*P value*
**Breakfast on weekdays**				
Never	Reference			
One day	0.98	0.97	0.99	0.002
Two days	0.98	0.97	0.99	0.001
Three days	0.97	0.96	0.98	<0.001
Four days	1.03	1.02	1.04	<0.001
Five days	1.22	1.22	1.23	<0.001
**Breakfast at weekends**				
Never	Reference			
One day	1.12	1.11	1.13	<0.001
Both days	1.41	1.40	1.42	<0.001
**Fruit consumption**				
Never	Reference			
Less than once a week	0.94	0.92	0.95	<0.001
Once a week	1.00	0.98	1.01	0.828
2-4 days a week	1.11	1.10	1.13	<0.001
5-6 days a week	1.27	1.25	1.28	<0.001
Once daily	1.34	1.33	1.36	<0.001
More than once daily	1.46	1.44	1.48	<0.001
**Vegetable consumption**				
Never	Reference			
Less than once a week	0.91	0.90	0.93	<0.001
Once a week	0.98	0.97	0.99	0.003
2-4 days a week	1.02	1.01	1.03	<0.001
5-6 days a week	1.06	1.05	1.08	<0.001
Once daily	1.08	1.06	1.09	<0.001
More than once daily	1.19	1.18	1.21	<0.001
**Sweet consumption**				
Never	1.09	1.07	1.10	<0.001
Less than once a week	1.08	1.07	1.09	<0.001
Once a week	1.07	1.06	1.08	<0.001
2-4 days a week	0.98	0.98	0.99	<0.001
5-6 days a week	0.97	0.96	0.98	<0.001
Once daily	1.04	1.03	1.05	<0.001
More than once daily	Reference			
**Soft drink consumption**				
Never	0.80	0.79	0.81	<0.001
Less than once a week	0.85	0.84	0.86	<0.001
Once a week	0.87	0.86	0.87	<0.001
2-4 days a week	0.89	0.89	0.90	<0.001
5-6 days a week	0.89	0.88	0.90	<0.001
Once daily	0.97	0.96	0.97	<0.001
More than once daily	Reference			

Note: Models were adjusted for sex, age, if the family was well-off, body mass index, physical activity, TV watching, playing computer games, computer use, smoking, and alcohol intake. OR: odds ratio; CI: confidence interval; Reference: reference group.

Table S6. Results of the linear model using life satisfaction as the outcome in boys (higher scores indicate higher life satisfaction).

	Coefficient	95% CI		*p value*
**Breakfast on weekdays**				
Never	Reference			
One day	-0.01	-0.02	0.00	0.080
Two days	0.01	0.00	0.02	0.026
Three days	-0.01	-0.01	0.00	0.180
Four days	-0.02	-0.03	-0.01	<0.001
Five days	0.17	0.16	0.17	<0.001
**Breakfast at weekends**				
Never	Reference			
One day	0.10	0.09	0.10	<0.001
Both days	0.25	0.25	0.26	<0.001
**Fruit consumption**				
Never	Reference			
Less than once a week	-0.08	-0.09	-0.07	<0.001
Once a week	0.02	0.01	0.03	<0.001
2-4 days a week	0.09	0.08	0.10	<0.001
5-6 days a week	0.17	0.16	0.18	<0.001
Once daily	0.22	0.21	0.23	<0.001
More than once daily	0.28	0.27	0.29	<0.001
**Vegetable consumption**				
Never	Reference			
Less than once a week	0.03	0.02	0.04	<0.001
Once a week	0.06	0.05	0.07	<0.001
2-4 days a week	0.10	0.10	0.11	<0.001
5-6 days a week	0.13	0.12	0.14	<0.001
Once daily	0.12	0.11	0.13	<0.001
More than once daily	0.12	0.11	0.13	<0.001
**Sweet consumption**				
Never	0.00	0.00	0.01	0.399
Less than once a week	-0.01	-0.02	-0.01	<0.001
Once a week	0.00	0.00	0.01	0.192
2-4 days a week	-0.03	-0.03	-0.02	<0.001
5-6 days a week	-0.01	-0.02	-0.01	<0.001
Once daily	-0.03	-0.03	-0.02	<0.001
More than once daily	Reference			
**Soft drink consumption**				
Never	-0.04	-0.05	-0.03	<0.001
Less than once a week	-0.10	-0.11	-0.10	<0.001
Once a week	-0.05	-0.05	-0.04	<0.001
2-4 days a week	-0.07	-0.08	-0.07	<0.001
5-6 days a week	-0.05	-0.06	-0.04	<0.001
Once daily	0.00	-0.01	0.01	0.996
More than once daily	Reference			

Note: Models were adjusted for sex, age, if the family was well-off, body mass index, physical activity, TV watching, playing computer games, computer use, smoking, and alcohol intake. CI: confidence interval; Reference: reference group.

Table S7. Results of the linear model using life satisfaction as the outcome in girls (higher scores indicate higher life satisfaction).

	Coefficient	95% CI		*p value*
**Breakfast on weekdays**				
Never	Reference			
One day	0.05	0.04	0.06	<0.001
Two days	0.02	0.01	0.03	<0.001
Three days	0.05	0.04	0.06	<0.001
Four days	0.06	0.05	0.07	<0.001
Five days	0.29	0.29	0.30	<0.001
**Breakfast at weekends**				
Never	Reference			
One day	0.24	0.23	0.25	<0.001
Both days	0.47	0.46	0.47	<0.001
**Fruit consumption**				
Never	Reference			
Less than once a week	-0.12	-0.13	-0.10	<0.001
Once a week	0.03	0.01	0.04	<0.001
2-4 days a week	0.06	0.05	0.07	<0.001
5-6 days a week	0.15	0.14	0.16	<0.001
Once daily	0.18	0.17	0.19	<0.001
More than once daily	0.27	0.26	0.28	<0.001
**Vegetable consumption**				
Never	Reference			
Less than once a week	0.10	0.09	0.11	<0.001
Once a week	0.12	0.11	0.13	<0.001
2-4 days a week	0.15	0.14	0.16	<0.001
5-6 days a week	0.17	0.17	0.18	<0.001
Once daily	0.17	0.16	0.18	<0.001
More than once daily	0.21	0.20	0.22	<0.001
**Sweet consumption**				
Never	-0.17	-0.18	-0.16	<0.001
Less than once a week	-0.10	-0.11	-0.09	<0.001
Once a week	-0.08	-0.09	-0.07	<0.001
2-4 days a week	-0.11	-0.11	-0.10	<0.001
5-6 days a week	-0.09	-0.09	-0.08	<0.001
Once daily	0.00	-0.01	0.00	0.158
More than once daily	Reference			
**Soft drink consumption**				
Never	-0.08	-0.09	-0.07	<0.001
Less than once a week	-0.07	-0.07	-0.06	<0.001
Once a week	-0.07	-0.08	-0.06	<0.001
2-4 days a week	-0.09	-0.10	-0.09	<0.001
5-6 days a week	-0.09	-0.10	-0.08	<0.001
Once daily	-0.04	-0.04	-0.028	<0.001
More than once daily	Reference			

Note: Models were adjusted for sex, age, if the family was well-off, body mass index, physical activity, TV watching, playing computer games, computer use, smoking, and alcohol intake. CI: confidence interval. Reference: reference group.

Table S8. Results of the linear model using life satisfaction as the outcome in 11-year-olds (higher scores indicate higher life satisfaction).

	Coefficient	95% CI		*p value*
**Breakfast on weekdays**				
Never	Reference			
One day	0.11	0.10	0.12	<0.001
Two days	0.02	0.00	0.03	0.005
Three days	0.05	0.04	0.06	<0.001
Four days	-0.01	-0.02	0.01	0.342
Five days	0.23	0.22	0.24	<0.001
**Breakfast at weekends**				
Never	Reference			
One day	0.11	0.10	0.12	<0.001
Both days	0.30	0.29	0.31	<0.001
**Fruit consumption**				
Never	Reference			
Less than once a week	-0.09	-0.10	-0.07	<0.001
Once a week	0.11	0.09	0.12	<0.001
2-4 days a week	0.09	0.08	0.10	<0.001
5-6 days a week	0.19	0.18	0.21	<0.001
Once daily	0.26	0.25	0.27	<0.001
More than once daily	0.33	0.32	0.34	<0.001
**Vegetable consumption**				
Never	Reference			
Less than once a week	0.04	0.03	0.05	<0.001
Once a week	0.08	0.07	0.09	<0.001
2-4 days a week	0.11	0.10	0.12	<0.001
5-6 days a week	0.12	0.11	0.13	<0.001
Once daily	0.10	0.09	0.11	<0.001
More than once daily	0.13	0.12	0.14	<0.001
**Sweet consumption**				
Never	0.03	0.02	0.04	<0.001
Less than once a week	0.01	0.01	0.02	0.002
Once a week	0.00	-0.01	0.01	0.951
2-4 days a week	-0.04	-0.04	-0.03	<0.001
5-6 days a week	-0.01	-0.02	0.00	0.002
Once daily	0.00	-0.01	0.01	0.767
More than once daily	Reference			
**Soft drink consumption**				
Never	-0.01	-0.02	0.00	0.022
Less than once a week	-0.09	-0.10	-0.09	<0.001
Once a week	-0.03	-0.04	-0.03	<0.001
2-4 days a week	-0.10	-0.11	-0.09	<0.001
5-6 days a week	-0.05	-0.06	-0.04	<0.001
Once daily	-0.10	-0.11	-0.09	<0.001
More than once daily	Reference			

Note: Models were adjusted for sex, age, if the family was well-off, body mass index, physical activity, TV watching, playing computer games, computer use, smoking, and alcohol intake. CI: confidence interval; Reference: reference group.

Table S9. Results of the linear model using life satisfaction as the outcome in 13-year-olds (higher scores indicate higher life satisfaction).

	Coefficient	95% CI		*p value*
**Breakfast on weekdays**				
Never	Reference			
One day	0.05	0.04	0.06	<0.001
Two days	0.07	0.06	0.08	<0.001
Three days	0.02	0.01	0.03	<0.001
Four days	0.07	0.06	0.08	<0.001
Five days	0.28	0.27	0.29	<0.001
**Breakfast at weekends**				
Never	Reference			
One day	0.21	0.20	0.22	<0.001
Both days	0.44	0.43	0.45	<0.001
**Fruit consumption**				
Never	Reference			
Less than once a week	-0.17	-0.18	-0.15	<0.001
Once a week	-0.04	-0.05	-0.03	<0.001
2-4 days a week	0.01	0.00	0.03	0.011
5-6 days a week	0.12	0.11	0.13	<0.001
Once daily	0.13	0.11	0.14	<0.001
More than once daily	0.20	0.19	0.22	<0.001
**Vegetable consumption**				
Never	Reference			
Less than once a week	0.01	0.00	0.02	0.224
Once a week	0.04	0.03	0.05	<0.001
2-4 days a week	0.10	0.09	0.11	<0.001
5-6 days a week	0.11	0.10	0.12	<0.001
Once daily	0.14	0.13	0.15	<0.001
More than once daily	0.15	0.13	0.16	<0.001
**Sweet consumption**				
Never	-0.09	-0.10	-0.08	<0.001
Less than once a week	-0.07	-0.07	-0.06	<0.001
Once a week	-0.05	-0.06	-0.04	<0.001
2-4 days a week	-0.06	-0.07	-0.06	<0.001
5-6 days a week	-0.02	-0.03	-0.02	<0.001
Once daily	0.00	0.00	0.01	0.201
More than once daily	Reference			
**Soft drink consumption**				
Never	-0.08	-0.09	-0.08	<0.001
Less than once a week	-0.09	-0.10	-0.08	<0.001
Once a week	-0.07	-0.07	-0.06	<0.001
2-4 days a week	-0.09	-0.10	-0.09	<0.001
5-6 days a week	-0.09	-0.10	-0.08	<0.001
Once daily	-0.01	-0.02	0.00	0.059
More than once daily	Reference			

Note: Models were adjusted for sex, age, if the family was well-off, body mass index, physical activity, TV watching, playing computer games, computer use, smoking, and alcohol intake. CI: confidence interval; Reference: reference group.

Table S10. Results of the linear model using life satisfaction as the outcome in 15-year-olds (higher scores indicate higher life satisfaction).

	Coefficient	95% CI		*P value*
**Breakfast on weekdays**				
Never	Reference			
One day	-0.06	-0.07	-0.05	<0.001
Two days	-0.02	-0.02	-0.01	0.001
Three days	0.04	0.03	0.05	<0.001
Four days	0.04	0.03	0.05	<0.001
Five days	0.21	0.21	0.22	<0.001
**Breakfast at weekends**				
Never	Reference			
One day	0.14	0.13	0.15	<0.001
Both days	0.31	0.30	0.31	<0.001
**Fruit consumption**				
Never	Reference			
Less than once a week	-0.07	-0.08	-0.05	<0.001
Once a week	-0.01	-0.02	0.00	0.192
2-4 days a week	0.09	0.08	0.10	<0.001
5-6 days a week	0.14	0.13	0.15	<0.001
Once daily	0.19	0.17	0.20	<0.001
More than once daily	0.28	0.27	0.29	<0.001
**Vegetable consumption**				
Never	Reference			
Less than once a week	0.14	0.13	0.16	<0.001
Once a week	0.13	0.12	0.14	<0.001
2-4 days a week	0.14	0.13	0.16	<0.001
5-6 days a week	0.19	0.18	0.20	<0.001
Once daily	0.16	0.15	0.17	<0.001
More than once daily	0.19	0.18	0.20	<0.001
**Sweet consumption**				
Never	-0.18	-0.20	-0.17	<0.001
Less than once a week	-0.14	-0.15	-0.13	<0.001
Once a week	-0.07	-0.08	-0.07	<0.001
2-4 days a week	-0.11	-0.11	-0.10	<0.001
5-6 days a week	-0.12	-0.13	-0.11	<0.001
Once daily	-0.05	-0.05	-0.04	<0.001
More than once daily	Reference			
**Soft drink consumption**				
Never	-0.10	-0.11	-0.09	<0.001
Less than once a week	-0.07	-0.07	-0.06	<0.001
Once a week	-0.10	-0.11	-0.09	<0.001
2-4 days a week	-0.07	-0.08	-0.06	<0.001
5-6 days a week	-0.06	-0.07	-0.05	<0.001
Once daily	0.03	0.03	0.04	<0.001
More than once daily	Reference			

Note: Models were adjusted for sex, age, if the family was well-off, body mass index, physical activity, TV watching, playing computer games, computer use, smoking, and alcohol intake. CI: confidence interval; Reference: reference group.
